# Cytochrome oxidase I DNA barcodes of crocodilians meat selling in Hong Kong

**DOI:** 10.1038/s41597-023-02889-3

**Published:** 2024-01-06

**Authors:** Wai Lok So, Tze Kiu Chong, Ivy Hoi Ting Lee, Miu Tsz Wai So, Avis Mang Yi Liu, Sam Tsz Chung Leung, Wai Ching, Ho Yin Yip, Pang Chui Shaw, Jerome Ho Lam Hui

**Affiliations:** 1 School of Life Sciences, The Chinese University of Hong Kong, Hong Kong SAR, China; 2 Simon F.S. Li Marine Science Laboratory, Institute of Environment, Energy and Sustainability, State Key Laboratory of Agrobiotechnology, The Chinese University of Hong Kong, Hong Kong SAR, China; 3 Li Dak Sum Yip Yio Chin R&D Centre for Chinese Medicine, The Chinese University of Hong Kong, Hong Kong SAR, China

**Keywords:** Conservation biology, Eukaryote

## Abstract

The crocodilians include true crocodiles, alligators, caimans, and gharial, and the trade of crocodilian products is regulated in accordance with the Convention of Wild Fauna and Flora (CITES). Hong Kong does not have her own wild crocodilians; thus, all crocodilians meat available is presumably imported with proper license. Here, we obtained a dataset of cytochrome oxidase I (COI) gene markers of 114 crocodilian meat samples (including frozen and dried crocodilian meat products) available in the contemporary market. We have also validated these barcodes in a phylogenetic approach with other data deposited on the GenBank, and detected 112 samples belonging to four crocodile species *Crocodylus siamensis*, *C. porosus*, *C. niloticus* and *Alligator mississippiensis*, and 2 samples belonging to snake *Malayopython reticulatus*. The dataset generated in this study will be useful for further studies including meat inspection, illegal trading, and enhancement of international and local legislations on illegal reptile importation.

## Background & Summary

The Crocodilia or Crocodylia is an order containing amphibious reptiles generally known as the crocodilians and can be found in the tropics, and certain areas in China and United States. The crocodilians include true crocodiles, alligators, caimans, and gharial, and the trade of crocodilian products is regulated in accordance with the CITES. For instance, commercial organisations such as alligator and crocodile farming need to demonstrate that they do not adversely impact the wild population. Nearly all parts of these animals are now traded, either as leather, meat, ornaments, and traditional Chinese medicine^1–6^.

The Convention on International Trade in Endangered Species of Wild Fauna and Flora (CITES) aims to protect endangered species from over-exploitation by controlling international trade through a permit system that ensures the survival of species. CITES have generated three appendices, namely CITES Appendix I, II and III, which are lists of species at various levels or kinds of protection from over-exploitation. According to the International Union for Conservation of Nature (IUCN) Red List of Threatened Species, 11 out of 23 extant crocodile species are currently listed as either critically endangered or vulnerable (https://www.iucnredlist.org), and 17 species are listed in the Appendix I of CITES, i.e. commercial trade is prohibited. Captive-bred of these species from CITES registered operations and artificially propagated for commercial purposes are considered as Appendix II specimens, and their trade is permitted with a valid license. To date, the crocodilian meat products that are available in the global markets are largely manufactured from the captive farms in Madagascar, South Africa, Zambia, Zimbabwe, Thailand and Vietnam, with *Crocodylus niloticus* and *C. siamensis* being the most cultured species^1^. Nevertheless, hunting and illegal trading continues to harm the wild population and their associated habitats^7^.

Hong Kong does not have wild crocodiles nor crocodilian farms, and thus all the crocodilian meat selling in the market for various purposes are basically imported with proper permits from crocodile farms^8–15^. CITES in Hong Kong is implemented through the enforcement of the Animals and Plants (Protection of Endangered Species) Ordinance, Cap 187, where no person shall import, export, or possess any endangered species, or parts and derivatives of such species, except under and in accordance with a license issued by the Director of Agriculture, Fisheries and Conservation Department, Hong Kong SAR Government. According to the data retrieved from CITES, crocodilian products imported into Hong Kong from 2018 to 2021 included 4 species including *Alligator mississipplensis* (CITES Appendix II)*, C. niloticus* (CITES Appendix I and II depending on the geographical locations, such as Botswana, Ethiopia, Kenya, Madagascar, Malawi, Mozambique, Namibia, South Africa, Uganda, the United Republic of Tanzania, Zambia and Zimbabwe)*, C. porosus* (CITES Appendix I and II depending on the geographical locations, such as populations of Australia, Indonesia, Malaysia, Papua New Guinea); and *C. siamensis* (CITES Appendix I).

From time to time, there are news of illegal importing of crocodiles to Hong Kong. In 2013, customs officers seized 579 kg of endangered crocodile meat hidden in a truck container^16^. More than a decade later in 2019, there was also report of endangered Southeast Asia species of crocodile at the Bride’s Pool presumably via illegal pet trade^17^. Another issue goes to the authenticity of crocodile meat being sold in Hong Kong. In 2006, a survey revealed only 10% of dry crocodile meat available in Hong Kong are genuine based on 11 samples^18^, and the Consumer Council of Hong Kong SAR Government further revealed only 30% of 24 samples were found to be unadulterated dried crocodile meat^19^. Understanding the contemporary situation of crocodile meat being sold in Hong Kong, will provide necessary up-to-date baseline information to further enhance the current practice in executing CITES and law enforcement.

**Fig. 1:**
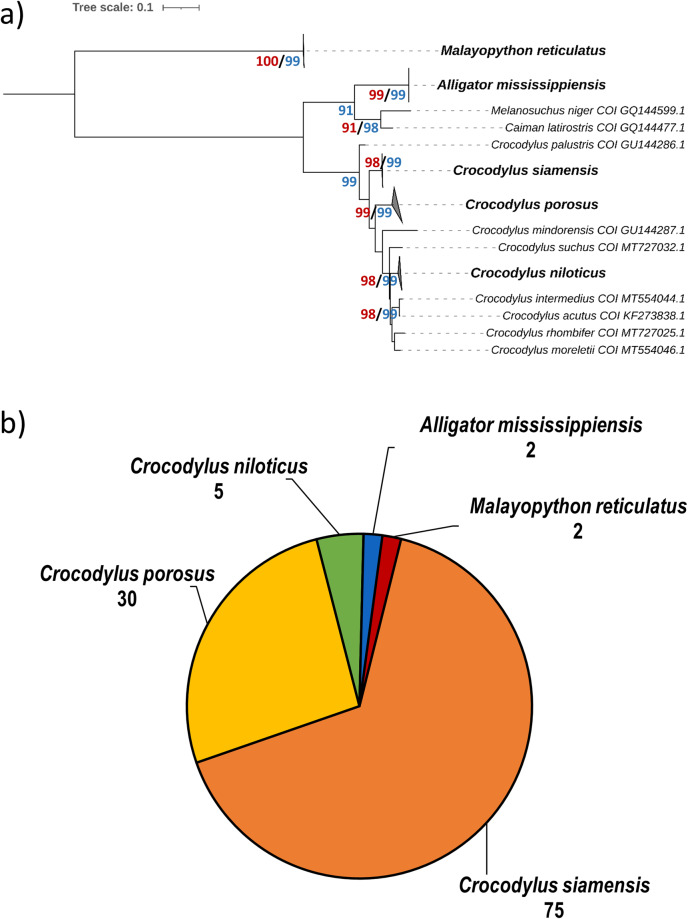
(**a**) Phylogenetic analysis of the sampled crocodilian meats using Maximum-Likelihood method (TIM + F + G4). Only bootstrap values equal to or greater than 90 were shown. (**b**) Summary of species identified in this study using COI marker.

In this study, a total of 114 samples including frozen and dried meat products were collected and identified at species level using cytochrome oxidase I (COI) gene markers. The COI sequences were then subjected to phylogenetic analysis for the validation of species identity (Fig. 1a, Supplementary Figure S1). Among the 114 investigated samples, 112 of them were crocodilians including *C. siamensis* (75), *C. porosus* (30), *C. niloticus* (5), and *A. mississipplensis* (2) (Supplementary Table S1 & S2), which agrees with the CITES Trade records^20^ (Fig. 1b). In addition, we also found that 2 samples were *Malayopython reticulatus*, which is a python species under CITES Appendix II (Fig. 1b).

## Methods

### Sampling and storage of samples

A total of 114 samples including 15 frozen and 99 dried claimed crocodile meat were purchased in Hong Kong, including 46 of them purchased from e-commerce platform while 68 were obtained from dried food or seafood stores in different geographical areas. Each of the samples were labelled with a corresponding voucher number for this study upon receival. A small portion of samples were removed from the package for DNA analysis, while the remaining tissues were properly stored. The dried samples were stored in a sealed plastic box with desiccant packs under room temperature, while the frozen samples were sealed in zipper bags and stored at -20 °C freezer. The Voucher numbers were assigned for each sample, and the metadata could be found in Supplementary Table S1.

### DNA extraction, PCR amplification of target markers and Sanger sequencing

All the equipment and consumables were autoclaved and cleaned with 75% ethanol, and disinfected under a flame prior to each sample experiment. Genomic DNA of the meat was isolated with a spin-column based extraction method using the PureLink™ Genomic DNA Mini Kit (Invitrogen, USA), following the manufacturer’s instructions. In brief, a small amount of tissue was excised from the package by a pair of disinfected scissors and placed in a 1.5 ml microcentrifuge tube. Tissue digestion buffer was then added into each tube and the tissue was homogenized thoroughly with autoclaved plastic homogenisers. The samples were then incubated in a dry bath with temperature set at 55 °C for 2–3 hours, or until the tissue completely disintegrated. The subsequent supernatant was obtained by centrifugation and subjected to ethanol precipitation, which was then passed through a spin-column to yield purified genomic DNA. A positive and negative (with no tissues) control tube was included in each extraction process.

The extracted DNA was subjected to quality and quantity control by 1% gel electrophoresis and One/OneC Microvolume UV-Vis Spectrophotometer (Thermo Scientific, NanoDrop, USA). The extracted DNA was kept in designated -20 °C freezer with cleared labels after sample preparation. The qualified genomic DNA was subjected to polymerase chain reaction (PCR) using a set of primer cocktails: (VF2_t1: 5′-TGTAAAACGACGGCCAGTCAACCAACCACAAAGACATTGGCAC-3′, FishF2_t1: 5′-TGTAAAACGACGGCCAGTCGACTAATCATAAAGATATCGGCAC-3′, FishR2_t1: 5′-CAGGAAACAGCTATGACACTTCAGGGTGACCGAAGAATCAGAA-3′ and FR1d_t1: CAGGAAACAGCTATGACACCTCAGGGTGTCCGAARAAYCARAA-3′) for amplifying mitochondrial cytochrome c oxidase subunit I (COI) gene^21^. For sample #41 and #113, where COI gene could not be amplified with the above primers, universal primers LCO1490/HCO2198 (LCO1490: 5′-GGTCAACAAATCATAAAGATATTGG-3′, HCO2198: 5′-TAAACTTCAGGGTGACCAAAAAATCA-3′)^22^ was used. PCR was carried out on a T100™ thermal cycler (Bio-Rad, USA) with the following parameters: an initial denaturation step at 95 °C for 3 minutes; followed by 36 amplification cycles of 30 seconds for denaturation at 95 °C, 30 seconds for primer annealing at 54 °C and 45 seconds for extension at 72 °C, and a final extension step at 72 °C for 5 minutes. The reaction mixture included PCR buffer, extracted genomic DNA sample, 2 mM dNTP, 1.5 mM MgCl_2_, 0.4 mM of each forward and reverse primers, and *Taq* DNA polymerase. A positive and negative (sample DNA is replaced by ddH_2_O) control tube was included in each PCR process. The amplified products were then validated by 1% agarose gel electrophoresis and sent to BGI Genomics Company Hong Kong for purification of PCR products by magnetic beads and Sanger sequencing on the platform ABI3730xl.

### Species identification and Phylogenetic analyses

After obtaining the DNA sequences, the chromatogram of each sample was carefully examined. If the chromatogram shows the limit in resolution, no clear peaks, or multiple sequences/peaks, the sequence was considered as not suitable for analysis. The corresponding sample was then proceeded to re-extraction, re-amplification, and re-sequencing. If any unincorporated detection of peaks, dye blob, pull up, mobility errors, or background noise were observed in the chromatogram, the sequence was manually edited, and the edited content was recorded.

Each resultant DNA sequence was compared to the non-redundant (nr) database on National Center for Biotechnology Information (NCBI) for homology analysis. Only sequences showing a similarity match > 98% to the annotated sequence on the sequence database were assigned to that species.

For phylogenetic analysis, the DNA sequences were aligned with reference crocodilian sequences on NCBI GenBank database using software MEGA7.0^23^. Neighbour-Joining (NJ) and Maximum-likelihood (ML) phylogenetic trees were constructed with 1,000 bootstraps in MEGA7.0^22^ and IQ-TREE ver. 2.0.3^24^ respectively. The trees were subsequently visualised and customized on iTOL tree^25^.

## Data Records

The dataset has been deposited on Genbank under the BioProject accession number PRJNA1053827^26^. An additional master file containing statistic information in this study is available on Figshare (10.6084/m9.figshare.23283839)^27^, which consists of the following parts:

1. A document of supplementary information includes Figure S1. Figure S1. Neighbor-joining phylogenetic tree of 114 crocodile samples purchased from Hong Kong.
2. An excel of Supplementary Tables S1-S2. Supplementary Table S1. Voucher and product details of crocodilian meat samples Supplementary Table S2. Species identity of crocodilian meat samples, COI sequences and the approved NCBI accession numbers.

## Technical Validation

### Sequence validation

The chromatogram of each sequence obtained from BGI was examined base by base on the software SnapGene Viewer. Manual deletion of primer sequences at the 5’ and 3’ ends were performed for each barcoded sequence.

### Species identification validation

Species identity of each sample was confirmed by phylogenetic analysis on COI marker gene. Only sequences having a similarity match > 98% to the annotated sequence on the database were assigned the identity.

## Usage Notes

Not applicable

## Data Availability

There was no custom code used in this study.
